# Immunological characterization and transcription profiling of peripheral blood (PB) monocytes in children with autism spectrum disorders (ASD) and specific polysaccharide antibody deficiency (SPAD): case study

**DOI:** 10.1186/1742-2094-9-4

**Published:** 2012-01-07

**Authors:** Harumi Jyonouchi, Lee Geng, Deanna L Streck, Gokce A Toruner

**Affiliations:** 1 Division of Allergy/Immunology and Infectious Diseases, Department of pediatrics, UMDN-NJMS, 185 South Orange Ave. Newark, NJ 07101-1709, USA; 2 The Institute of Genomic Medicine, Department of Pediatrics, UMDNJ-NJMS185 South Orange Ave. Newark, NJ 07101-1709, USA

**Keywords:** autism spectrum disorders (ASD), cytokine, innate immunity, transcription profiling, monocytes, specific polysaccharide antibody deficiency (SPAD)

## Abstract

**Introduction:**

There exists a small subset of children with autism spectrum disorders (ASD) characterized by fluctuating behavioral symptoms and cognitive skills following immune insults. Some of these children also exhibit specific polysaccharide antibody deficiency (SPAD), resulting in frequent infection caused by encapsulated organisms, and they often require supplemental intravenous immunoglobulin (IVIG) (ASD/SPAD). This study assessed whether these ASD/SPAD children have distinct immunological findings in comparison with ASD/non-SPAD or non-ASD/SPAD children.

**Case description:**

We describe 8 ASD/SPAD children with worsening behavioral symptoms/cognitive skills that are triggered by immune insults. These ASD/SPAD children exhibited delayed type food allergy (5/8), treatment-resistant seizure disorders (4/8), and chronic gastrointestinal (GI) symptoms (5/8) at high frequencies. Control subjects included ASD children without SPAD (N = 39), normal controls (N = 37), and non-ASD children with SPAD (N = 12).

**Discussion and Evaluation:**

We assessed their innate and adaptive immune responses, by measuring the production of pro-inflammatory and counter-regulatory cytokines by peripheral blood mononuclear cells (PBMCs) in responses to agonists of toll like receptors (TLR), stimuli of innate immunity, and T cell stimulants. Transcription profiling of PB monocytes was also assessed. ASD/SPAD PBMCs produced less proinflammatory cytokines with agonists of TLR7/8 (IL-6, IL-23), TLR2/6 (IL-6), TLR4 (IL-12p40), and without stimuli (IL-1ß, IL-6, and TNF-α) than normal controls. In addition, cytokine production of ASD/SPAD PBMCs in response to T cell mitogens (IFN-γ, IL-17, and IL-12p40) and candida antigen (Ag) (IL-10, IL-12p40) were less than normal controls. ASD/non-SPAD PBMDs revealed similar results as normal controls, while non-ASD/SPAD PBMCs revealed lower production of IL-6, IL-10 and IL-23 with a TLR4 agonist. Only common features observed between ASD/SPAD and non-ASD/SPAD children is lower IL-10 production in the absence of stimuli. Transcription profiling of PB monocytes revealed over a 2-fold up (830 and 1250) and down (653 and 1235) regulation of genes in ASD/SPAD children, as compared to normal (N = 26) and ASD/non-SPAD (N = 29) controls, respectively. Enriched gene expression of TGFBR (p < 0.005), Notch (p < 0.01), and EGFR1 (p < 0.02) pathways was found in the ASD/SPAD monocytes as compared to ASD/non-SPAD controls.

**Conclusions:**

The Immunological findings in the ASD/SPAD children who exhibit fluctuating behavioral symptoms and cognitive skills cannot be solely attributed to SPAD. Instead, these findings may be more specific for ASD/SPAD children with the above-described clinical characteristics, indicating a possible role of these immune abnormalities in their neuropsychiatric symptoms.

## Background

Mounting evidence indicate that ASD is a behaviorally defined syndrome associated with multiple genetic and environmental factors, resulting in similar behavioral symptoms [1-4]. The exceptions are small subsets of patients with known gene mutations (up to 15-20%) [5]. Consequently, ASD is characterized by varying clinical phenotypes and a high frequency of co-morbidities. These co-morbid conditions often have inflammatory components and inflammation and immune activation has been implicated in ASD pathogenesis [6,7]. However, previous studies addressing immune abnormalities in ASD children have been inconclusive, partly due to the marked heterogeneity of the study subjects.

Previously, we reported a subset of ASD children whose clinical symptoms are characterized by worsening behavioral symptoms and loss of once acquired cognitive skills triggered by benign immune insults, typically common childhood infection [8]. Among this subset of ASD children, designated as the ASD-test group in the previous study, we found a high frequency of immunodeficiency (mainly SPAD), requiring treatment of intravenous immunoglobulin (IVIG) [8]. SPAD is clinically characterized by impaired antibody production against encapsulated organisms that are common causes of pneumonia, sinusitis, and ear infection. Therefore, in the previous study, ASD/SPAD children were excluded from the further analysis, due to the concern that the presence of SPAD and resultant presence of active infection may affect the results of our immunological assays. Thus, we do not know whether ASD/SPAD children with fluctuations in behavioral symptoms/cognitive skills have the innate immune abnormalities observed in the ASD test group [8] or if they manifest immune abnormalities more specific for SPAD.

In the Pediatric Allergy/Immunology (A/I) Clinic at our institution, we follow 8 ASD/SPAD children who have worsening behavioral symptoms/cognitive skills with immune insults. In these ASD/SPAD children, even after improved control of infectious complications with IVIG, we still observe worsening behavioral symptoms/cognitive skills that are triggered by immune insults. These children also seem to have treatment-resistant seizure disorders at a higher frequency than the ASD/non-SPAD children. In our observation, there were no differences between ASD/SPAD children and non-ASD children with SPAD (non-ASD/SPAD) in the routine immune workups. Infectious complications observed in these ASD/SPAD children in our clinic were very similar to those observed in non-ASD/SPAD children [9]. Innate immune responses are not routinely studied in conventional immune workups for SPAD. Since our previous studies have indicated innate immune abnormalities in the ASD test group children [8], we hypothesized that innate immune responses affecting the development of adaptive cellular and humoral immunity are altered in the ASD/SPAD children who reveal worsening behavioral symptoms and cognitive skills with immune insults. We also hypothesized that these altered immune responses are not attributed to SPAD but are associated with their characteristic neuropsychiatric symptoms as described above, perhaps reflecting impaired neuroimmune network.

In this study, we tested our hypotheses by further characterizing 8 ASD/SPAD children with fluctuating behavioral symptoms/cognitive skills, by analyzing their clinical features and immunological findings in comparison with three control groups: ASD/non-SPAD children, normal control children, and non-ASD/SPAD children. The obtained results support our initial hypothesis, that peripheral blood mononuclear cells (PBMCs) from ASD/SPAD children reveal distinct innate and adaptive immune abnormalities not shared by ASD/non-SPAD or non-ASD/SPAD children.

### Case description

#### ASD/SPAD children

Eight ASD/SPAD children characterized by fluctuating (worsening) behavioral/cognitive skills following immune insults including viral infection and adverse reactions to medications were presented in this study. The demographics, diagnosis, co-morbidities, and clinical laboratory findings of these ASD/SPAD children are summarized in Tables 1 and 2. These ASD/SPAD children had at least 3 occurrences of worsening behavioral symptoms and/or loss of once acquired cognitive skills documented following immune insults such as viral syndrome; viral syndrome (upper respiratory infection and acute gastroenteritis) was clinically diagnosed with negative streptococcal antigen in throat swab and in some cases, supported by positive viral antigen and/or DNA in nasal secretions by PCR. Occurrences of worsening behavioral symptoms were independently documented by caretakers, teachers, and therapists. SPAD was diagnosed with detectable antibody (Ab) titers (> 1.0 μg/ml) to less than 3 of 14 serotypes of *Streptococcus pneumonia *tested in response to Pneumovax^® ^[10], a standard diagnostic measure for SPAD. All of the ASD/SPAD children evaluated in this study are currently on IVIG (0.6-1 g/kg/dose every 3 weeks), since their infection complications were not controlled effectively with prophylactic antibiosis. Assay samples were obtained when their infectious complications were well under control after implementation of IVIG treatment. The ages of ASD/SPAD children at the time of sample obtainment were 12.3 yr (median) with range of 8.3-17.5 yr. The length of IVIG treatment varied from 1 to 6 yrs, at the time blood samples were obtained. It should be noted that we also follow 2 ASD/SPAD children without fluctuation in behavioral symptoms/cognitive skills. They responded very well to IVIG treatment for controlling infections but we did not observe any changes in their autistic features. These children were not included in the current study. We refer ASD/SPAD children as these 8 ASD/SPAD children with worsening behavioral symptoms and cognitive skills with immune insults in this study.

**Table 1:** Demographics and clinical features of the ASD/SPAD children

Case	Age^1 ^(yr)	Race	Sex	Immuno-deficiency Diagnosis	Autism Diagnosis	Infection	Other co-morbidities and medications^3^
#1^4^	13	W	M	SPAD^5^	Regressive autism	CRS, ROM	Chronic enterocolitis, asthma fluoxetine, montelukast,

#2^4^	11	W	M	SPAD	Regressive autism	CRS, ROM	Seizure disorder^7^, Chronic enterocolitis levetiracetam, montelukast, loratadine,

#3^4^	8	W	M	SPAD	Regressive autism	CRS	Chronic enterocolitis, allergic rhiniconjunctivitis fluoxetine, montelukast, cetirizine, mometasone nasal inhaler

#4^4^	9	W	M	SPAD	PDD-NOS (regressive)	ROM	Seizure disorder valproic acid, L-carnitine, CQ10

#5^2,4^	14	W	F	SPAD	Regressive autism	CRS, ROM,	Seizure disorder, Chronic enterocolitis, asthma montelukast, desloratadine, minocycline (for acne), lorazepam

#6	16	W	M	SPAD	Regressive autism	CRS	Asthma, chronic enterocolitis Steroid oral inhaler, nasal inhaler, guanfacine

#7	7	W	F	SPAD	PDD-NOS (regressive)	CRS, ROM	Seizure disorder montelukast, nasal inhaler, levetiracetam, azithromycin (prophylaxis)

#8	6	mixed	M	SPAD^6^	Regressive autism	COM	guanfacine, risperidone, benzatropine

^1 ^Ages at the time of SPAD diagnosis. It should be noted that Case #1 and Case #2 were followed up in the clinic for 2-3 yrs prior to SPAD diagnosis but their initial laboratory values were not consistent with SPAD diagnosis. Their clinical features progressed over 2-3 yrs to fulfill the diagnosis of SPAD.

^2 ^This patient developed anti-phospholipid syndrome 5 yrs after being treated with IVIG.

^3 ^Co-morbidities present at the time of presentation and medications at the time of sample obtainment.

^4 ^Positive history of food protein induced enterocolitis syndrome (FPIES)

^5 ^Abbreviations used: COM (chronic otitis media), CRS (chronic rhinosinusitis), PDD-NOS (pervasive developmental disorder, not otherwise specified), ROM (recurrent otitis media), SPAD (specific polysaccharide deficiency), and W (Caucasians)

^6 ^This patient also revealed low IgG levels but did not fall into the diagnostic criteria for common variable immunodeficiency; immunoglobulin levels of 2 isotypes are lower than two standard deviations of mean values of age-appropriate controls.

^7 ^In Case #2 and Case #7, a main trigger of seizure activities has been respiratory infection. In Case#7, onset of seizure clusters were almost always triggered by respiratory infection prior to IVIG treatment. After implementation of IVIG treatment, no clinical seizures have been observed in case #2. In case #7, seizure activity appears to be not associated with infection any more after starting IVIG treatment and prophylaxis doses of azithromycin (3 times per week).

**Table 2:** Summary of conventional immune workup results in ASD/SPAD children

Case#	IgG^1 ^(mg/dL)	IgA (mg/dL)	IgM (mg/dL)	Pneumococcal antibody titers^2 ^*N > 11/14*	Isotype-switched memory B cells (cells/μl)^3^ *N *= *39.9 (27.1-97.6)*^*4*^	Total memory B cells (cells/μl) *N *= *77.5 (55.1-198)*^*4*^
#1	545	23	96	0/14	7.6	19.6

#2	844	116	190	0/14	8.3	13.2

#3	680	35	23	0/14	6.1	15.1

#4	767	34	59	1/14	5.4	25.0

#5	578	121	136	0/14	2.7	2.9

#6	783	40	107	0/14	21.9	31.0

#7	700	38	29	2/14	8.0	40.8

#8	442	44	66	2/14	1.9	19.0

^1 ^Normal ranges of Ig levels (mg/dl) are for children of 6-8 yr (IgG; 572-1374, IgM; 30-208, IgA; 34-305) for children of 9-11 yr (IgG; 586-1496, IgM; 48-228, IgA; 45-305) and for children of 12-16 yr (IgG 759-1549, IgM; 35-239, IgA; 58-358).

^2 ^Numbers of serotypes revealed > 1.0 μg/ml Ab levels among *Streptococcus pneumonia *14 serotypes tested. Children > 5 yr is expected to reveal protective levels of Ab titers > 75% of 14 serotype.

^3 ^Isotype switched memory B cell numbers identified as IgD^-^, CD27^+^, CD19^+ ^cells and total memory B cell numbers (CD27^+^, CD19^+ ^cells) were expressed as cell numbers/μl of peripheral blood.

^4 ^Reference values of these parameters obtained from 27 normal controls (Age; 2-17 yr) in our laboratory are shown as a median (range).

In both ASD/SPAD and ASD/non-SPAD children, autism diagnosis was from established autism diagnostic centers, including the center at our institution. Standard diagnostic measures, including ADOS (Autism Diagnostic Observational Schedules), and ADI-R (Autism Diagnostic Interview-Revised) were used. Diagnosis of allergic disorders and asthma were based on diagnostic criteria described elsewhere [11-13].

#### Control subjects

1. *ASD/non-SPAD children*: These children were recruited in the Subspecialty Clinic at UMDNJ-NJMS where subspecialties include allergy/immunology, cardiology, developmental pediatrics, endocrinology, gastroenterology, genetics, general pediatrics, nephrology, and pulmonology. A total of 39 ASD/non-SPAD children were recruited to the study: 4 females and 35 males, median age: 8.1 yr, range; 5-17 yr, 7 African Americans (AA), 3 Asians, 27 Caucasians (W), and 2 mixed races. These ASD/non-SPAD children were diagnosed with autism (N = 25) or PDD-NOS (N = 14). Twenty out of 39 children reported to have developmental regression at the time of initial diagnosis of ASD. Allergic rhinoconjunctivitis (AR+AC) was diagnosed in 5/39 (12.8%) ASD/non-SPAD children. None of the control ASD/non-SPAD children were documented to have recurrent infection and/or fluctuating behavioral symptoms/cognitive skills following immune insults. In addition, none of these ASD/non-SPAD controls were diagnosed with seizure disorders.

2. *Normal controls*: Normal control children (N = 37, 8 females and 29 males, median age; 10.2 yr, range: 5-17 yr, 4AA, 1 Asian, 31 W, and 1 mixed race) were recruited in the Pediatric Subspecialty Clinic. In most cases, blood samples were obtained when they were medically indicated to have venipuncture for general health screening.

3. *Non-ASD/SPAD children*: A total of 12 non-ASD/SPAD children were recruited in the pediatric Allergy/Immunology Clinic; median age; 13.0 yr, range; 6-17 yr, 6 females and 6 males, 4 AA and 8 W. SPAD diagnosis was made as described in ASD/SPAD children in the previous section [10]. All of them have been treated with IVIG (0.6-1 g/kg/dose every 3 weeks) and were on IVIG treatment at the time of sample obtainment. The length of IVIG treatment was 1 to 7 yrs for these children at the time of this study. Two out of 12 patients were diagnosed with seizure disorders; in 1 patient, the seizure disorder was attributed to sequel of intracranial abscess developed from chronic rhinosinusitis and in 1 patient, the etiology of seizure disorder is unknown.

##### Sample obtainment

The study subjects were recruited following study protocols approved by the Institutional Review Board, University of Medicine and Dentistry of New Jersey-New Jersey Medical School (UMDNJ-NJMS). Blood samples were collected after obtainment of signed parental consent forms. Signed assent forms were also obtained, if applicable, in children older than 7 years of age.

At the time of sample obtainment, all the subjects were examined to ensure absence of active infection. Assays for adaptive and innate immunity for SPAD children, with or without ASD, were conducted after their conditions were stabilized by IVIG and became free from active infection. In most cases, sample obtainment coincided with medically indicated blood work.

##### Clinical and laboratory findings in ASD/SPAD children by conventional immune workup

Most of the ASD/SPAD children suffered from chronic rhinosinusitis (CRS) and recurrent otitis media (ROM) requiring frequent antibiosis prior to IVIG treatment. Four out of 8 (50%) ASD/SPAD children had history of multiple placements of pressure equalizing (PE) tubes bilaterally (Table 1). ASD/SPAD children also revealed a high frequency of treatment-resistant seizure disorders (4/8, 50%), while none of the normal and ASD/non-SPAD control children had a history of seizure disorders. However, 25/40 (62.5%) ASD/non-SPAD children had a history of food protein induced enterocolitis syndrome (FPIES), although at the time of blood sampling, none of them had active GI symptoms. ASD/SPAD children also had history of FPIES at a similar rate (5/8, 62.5%) and these ASD/SPAD children with history of FPIES suffered from chronic enterocolitis, even after IVIG treatment, requiring dietary intervention measures (avoidance of offending food). In these ASD/SPAD children with GI symptoms, extensive workups ruled out chronic microbial infection, celiac disease, inflammatory bowel diseases, or other well established GI diseases by endoscopic and histological examinations. None of the ASD/SPAD subjects revealed positive allergy workups; they all had normal IgE levels, negative reactivity to prick skin testing (PST), or negative for food allergen specific IgE. All the ASD/SPAD children were diagnosed with regressive type ASD (Table 1).

As shown in Table 2, immune workups, at initial diagnosis of SPAD, revealed low to low normal serum levels of IgG, IgA, and IgM as compared to age-appropriate controls in 4/8, 6/8, and 6/8 ASD/SPAD children. Numbers of isotype-switched memory B cell were lower than 10/μl in 7/8 ASD/SPAD children [14]. Infection was better controlled after initiation of IVIG treatment in all the ASD/SPAD children, which was also associated with a reduction in frequency of worsening behavioral symptoms triggered by infection. After being treated with several doses of IVIG, the parents of one patient reported the return of cognitive skills that were present prior to major regression. However, 7/8 ASD/SPAD children did not reveal significant improvement in autism behavioral symptoms or cognitive activity, per parental reports assessed by the clinical global impression scale (CGI) [15]. In the ASD/SPAD patients with seizure disorders, frequency of seizures was reduced after starting IVIG treatment, which is likely attributed to better control of infection, since the major trigger of seizure activity is infection for these subjects.

#### Further Evaluation of immune functions

##### 1. PBMC Cultures

PBMCs were isolated by Ficoll-Hypaque density gradient centrifugation. Innate immune responses were assessed by incubating PBMCs (10^6 ^cells/ml) overnight with TLR4 agonist (LPS; 0.1 μg/ml, GIBCO-BRL, Gaithersburg, MD), TLR2/6 agonist (zymosan; 50 μg/ml, Sigma-Aldrich, St. Luis, Mo), TLR3 agonist (Poly I:C, Poly I:C, 0.1 μg/ml, Sigma-Aldrich), and TLR7/8 agonist (CL097, water-soluble derivative of imidazoquinoline, 20 μM, InvivoGen, San Diego, CA) in RPMI 1640 with additives as previously described [8]. Overnight incubation was adequate to induce the optimal responses in this setting. Levels of proinflammatory [tumor necrosis factor-α (TNF-α), IL-1β, IL-6, IL-12p40, and IL-23] and counter-regulatory [IL-10, transforming growth factor-ß (TGF-ß) and soluble TNF receptor II (sTNFRII)] cytokines in culture supernatant were then measured by an enzyme-linked immunosorbent assay (ELISA).

Cellular reactivity to T cell stimulants was assessed by incubating PBMCs (10^6 ^cells/ml) with T cell mitogens [Con A (2 μg/ml) and PHA (5 μg/ml)], recall Ag [candida Ag (5 μg/ml), Greer, Lenoir, NC], and IFN-γ inducing cytokines [IL-12p70 (0.2 ng/ml, BD Biosciences, San Diego, CA), IL-18 (1 ng/ml, BD Biosciences) for 4 days and measuring levels of IFN-γ, TNF-α, IL-5, IL-10, IL-12p40, and IL-17 in the culture supernatant [8]. Initial titration studies showed that a four-day incubation period resulted in optimal production of these cytokines, in this setting.

Cytokine levels were measured by ELISA, using OptEIA™ Reagent Sets (BD Biosciences) for IFN-γ, IL-1ß, IL-5, IL-6, IL-10, IL-12p40, and TNF-α, and ELISA reagent set (R & D, Minneapolis, MN) for sTNFRII, IL-17 (IL-17A), and TGF-ß. IL-23 ELISA kit was purchased from eBiosciences, San Diego, CA. Intra- and inter-variations of cytokine levels were less than 5%.

##### 2. Flow cytometry

Memory B cells (IgD^-^, CD27^+^, CD19^+ ^B cells) were detected by staining with anti-CD45-FITC, anti-CD19-APC-Cy7, CD27-APC (all from BD biosciences, San Jose CA) and IgD-PE (DAKO, Carpinteria CA) monoclonal antibodies [14]. For intracellular cytokine staining in CD4^+ ^T cells, the following fluorochrome-conjugated monoclonal antibodies were used: CD4-PerCp, IFN-γ-PE-Cy7, IL-17-PE, IL-4-FITC, IL-10-Pacific Blue (all from eBiosicences), and TGF-ß-APC (R & D, Minneapolis, MN). PBMCs were incubated overnight (16 h) at 37°C with medium alone, Staphylococcal enterotoxin B (5 μg/mL, Sigma-Aldrich), or candida Ag (5 μg/ml, Greer) in the presence of Brefeldin A (BFA; 5 μg/ml, Sigma-Aldrich), anti-CD28 (1 μg/ml, eBiosciences), and anti-CD49 (1 μg/ml, eBiosciences) in the same culture medium used for the cytokine production assay. Then PBMCs were permeabilized (permeabilization buffer, BD Biosciences) and stained with the above described antibodies [16]. All flow cytometry was conducted using FACS Caliber or FACSVantage SE TM (BD Biosciences) and the data were analyzed with the CellQuest software (BD Biosciences) and FlowJo (TreeStar, Ashland, OR).

##### 3. Transcription profiling

Peripheral blood (PB) monocytes were purified using an immuno-affinity column following the manufacturer's instructions (MACS monocytes isolation kit, Miltenyi Biotec, Auburn, CA). Total RNA were extracted by the RNA easy kit (Quiagen, Valencia, CA). RNA labeling and hybridizations on Agilent Human 4 × 44K arrays (Agilent, Lexington, MA) were done using the Agilent One-Color Microarray-Based Gene Expression Analysis Ver 5.5 protocol (Agilent). All slides were scanned by an Agilent Scanner and normalized numerical data were obtained by Agilent Feature extraction software 9.5.

##### 4. Statistical analysis

For comparison of test values with control values, a Wilcoxon rank sum test was used. For comparison of values of multiple groups, a Kruskall-Wallis test was used. A Chi square (χ^2^) test was used to examine the difference in frequency and correlation was tested using a linear regression analysis. These tests were performed using R.2.10.1 (R-Development Core Team 2009). A p value of < 0.05 was considered to be statistically significant. For the analysis of microarrays experiments, Gene Spring GX v11 software (Agilent) was used. After filtering for "present" calls in at least 20% of samples, fold change analysis were performed for group for comparisons on 26992 probes. Genes with at least a two-fold change, as compared to controls, are determined to be either up-regulated or down-regulated. Using a specific module of GeneSpring software (Agilent), pathways enrichment analysis was performed on those up- or down-regulated genes to see if there is a statistically significant enrichment (p < 0.05) for specific BioPax pathways.

##### 5. Cytokine production results

As stated above, the median ages of the study subjects at the time of sample obtainment are 12.3 yr for ASD/SPAD children, 8.1 yr for ASD/non-SPAD children, 10.2 yr for normal controls, and 13.0 yr for non-ASD/SPAD children. Ages of ASD/non-SPAD children were lower than ASD/SPAD and non-ASD/SPAD children (p < 0.05). It should be noted that innate immune responses, as opposed to adaptive immune responses, are not expected to change with age. This is mainly because innate immunity is regulated by germ-line coded genes and has little post-natal modifications, such as gene rearrangement.

###### Responses to TLR agonists

**Figure 1:**
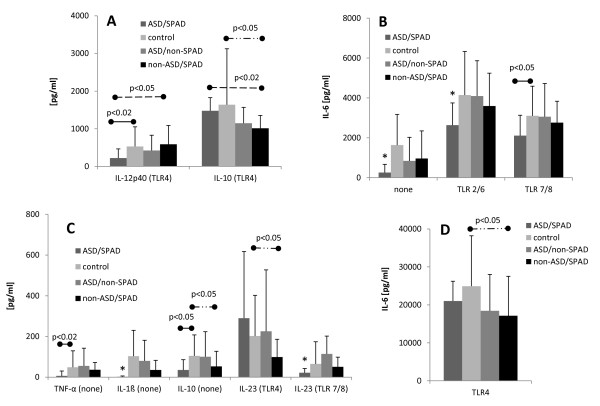
**Cytokine production by PBMCs from ASD/SPAD, normal control, ASD/non-SPAD and non-ASD/SPAD children when stimulated by TLR agonists**. IL-12p40/IL-10 production with TLR4 agonist (LPS) (Panel A), IL-6 production with TLR2/6 and 7/8 agonists or without a stimulus (Panel B), spontaneous production of TNF-α, IL-1ß, and IL-10 as well as IL-23 production with TLR4 and 7/8 agonists (Panel C), and IL-6 production with TLR4 agonist (Panel D) was shown. PBMCs (10^6 ^cells/ml) were incubated with TLR agonists as indicated overnight and cytokine levels in the culture supernatant were measured by ELISA. In Figs 1-2, the results of cytokine production with stimuli shown were those corrected by subtracting the levels of cytokines produced without a stimulus. *; lower than all the study groups (p < 0.05).

ASD/SPAD PBMCs produced different patterns of cytokine production. Namely, ASD/SPAD PBMCs produced lower amounts of IL-6 (without a stimulus and with TLR2/6 agonists), IL-1ß (without a stimulus), and IL-23 (with TLR 7/8 agonist) as compared to all the control groups (Figure 1 A, B, C). In, addition, ASD/SPAD PBMCs produced less IL-12p40 than normal and non-ASD/SPAD control cells in response to a TLR4 agonist (Figure 1-A). These cells also produced lower amounts of IL-6 (with the TLR 7/8 agonist) and TNF-α/IL-10 (in the absence of stimulus) than normal controls (Figure 1-B). PBMCs from ASD/non-SPAD children revealed similar patterns of cytokine production as compared to normal controls (Figure 1). In contrast, non-ASD/SPAD PBMCs revealed altered patterns of cytokine production which did not resemble those observed in ASD/SPAD children. That is, non-ASD/SPAD PBMC revealed lower IL-10 production than ASD/SPAD and normal control cells (Figure 1-A) and lower IL-6/IL-23 production than normal controls (Figure 1-C, D) in response to a TLR4 agonist. The only common feature observed between the ASD/SPAD and non-ASD/SPAD groups was production of lower levels of IL-10 in the absence of a stimulus, than normal controls (Figure 1-C). These results indicate that the altered responses to TLR agonists observed in the ASD/SPAD group are unlikely to be associated with SPAD.

###### Responses to the recall antigen and T cell mitogens

**Figure 2:**
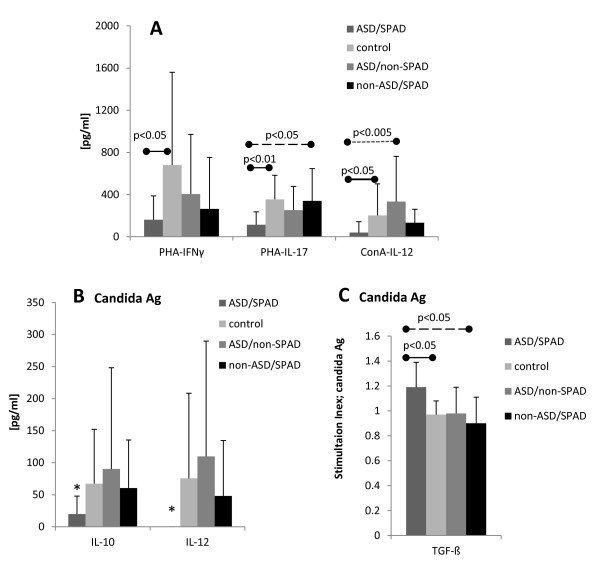
**Cytokine production by PBMCs from ASD/SPAD, normal control, ASD/non-SPAD and non-ASD/SPAD children when stimulated by mitogens (PHA or Con A) (Panel A) or candida Ag (Panels B and C)**. PBMCs (10^6 ^cells/ml) were incubated with T cell stimulants (PHA, Con A, and candida Ag) for 4 days and cytokine levels in the culture supernatant were measured by ELISA. Secondary to high production of TGF-ß without a stimulus, the production of TGF-ß is expressed as stimulation index (ratio of TGF-ß produced with candida Ag/background TGF-ß produced without a stimulus). *; lower than all the study groups (p < 0.05).

ASD/SPAD PBMCs also revealed altered patterns of T cell cytokine production as compared to control groups. Namely, ASD/SPAD PBMCs produced less IL-12p40 and IL-10 than all the study groups in response to a recall Ag (candida Ag) (Figure 2-B). In addition, ASD/SPAD PBMCs produced less IFN-γ and IL-17A with PHA, and less IL-12 with Con A as compared to normal controls (Figure 2-A). Moreover, amounts of IL-17A produced by ASD/SPAD PBMCs with PHA was also lower than that of non-ASD/SPAD cells (Figure 2-A). IL-12 production with Con A by ASD/SPAD cells was also lower than that of ASD/non-SPAD controls (Figure 2-A). Production of T cell cytokines by ASD/non-SPAD and non-ASD/SPAD cells did not differ from normal controls in response to T cell mitogens or candida Ag (Figure 2-A &2B). TGF-ß is produced in high amounts spontaneously and does not increase much in response to T cell stimuli. However, interestingly, we observed a higher increase in TGF-ß production in response to candida Ag in the ASD/SPAD group than normal and non-ASD/SPAD groups. Again, these results indicate that altered patterns of T cell cytokine production by the ASD/SPAD PBMCs is unlikely to be attributed to SPAD.

**Figure 3:**
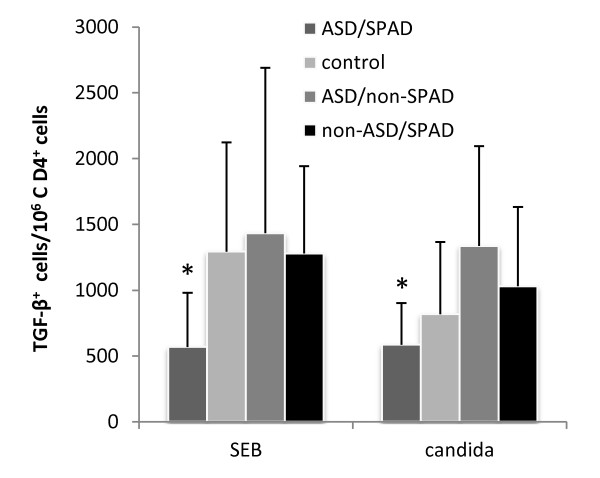
**Frequency of TGF-ß^+ ^CD4^+ ^cells/10^6 ^CD4^+ ^cells in the ASD/SPAD, normal control, ASD/non-SPAD, and non-ASD/SPAD children**. PBMCs were stimulated overnight with either SEB or candida Ag as described in the materials and methods section. *; lower than all the study groups (p < 0.05).

When intracellular expression of Th1 (IFN-γ), Th2 (IL-4), Th17 (IL-17A), and regulatory cytokines (IL-10 and TGF-ß) were examined in 6/8 ASD/SPAD children, we observed lower intracellular expression of TGF-β in CD4^+ ^cells, as compared to age-appropriate controls (ASD/non-SPAD children N = 18, normal controls N = 26, and non-ASD/SPAD children N = 9) (Figure 3).

##### 6. Transcription profiling of PB monocytes

Since most notable changes were found in TLR responses in ASD/SPAD children and these changes were not observed in non-ASD/SPAD controls, we conducted transcription profiling of PB monocytes, a major cell population of PB innate immune cells responding to TLR agonists. Transcription profiling was conducted in 7 ASD/SPAD, 28 ASD/non-SPAD, and 26 normal control children. We were unsuccessful in 2 our attempts to purify total RNA from PB monocytes of 1 ASD/SPAD child; this may be associated with the high dose of valproic acid that he was on in order to control his seizure activities. Our results revealed that significant numbers of genes were either up or down-regulated (> 2 fold) in ASD/SPAD monocytes as compared to controls, as summarized in Table 3. Up-regulated genes as compared to control groups included chemokines (CCL2 and CCL7). Pathway analysis of gene expression profiles revealed that ASD/SPAD children had enriched expression of genes involved in TGFBR (TGF-ß receptor), EGFR (epidermal growth factor receptor), and NOTCH pathways as compared to ASD/non-SPAD controls (Table 3).

**Table 3:** Summary of transcription profiling results in ASD/SPAD children

Numbers of genes^1^	Vs. normal controls	Vs. ASD/non-SPAD controls	Overlapping in 2 control groups
Up-regulated	830	1258	316

Down-regulated	653	1235	356

Pathway analysis	Vs. normal controls	Vs. ASD/non-SPAD controls	

TGFBR	Not significant	P < 0.01	

EGFR	Not significant	P < 0.02	

NOTCH	Not significant	P < 0.005	

^1 ^The number of genes > 2 fold up- or down-regulated in the ASD/SPAD children as compared to normal controls and ASD/non-SPAD children.

## Discussion and Evaluation

Clinical features of infection found in ASD/SPAD children are similar to those found in non-ASD/SPAD children and all of the ASD/SPAD children suffered from frequent sino-pulmonary infection (Table 2) [17]. As expected, infections were better controlled, once IVIG treatment was in place [18,19]. However, the ASD/SPAD children revealed a higher frequency of seizure disorders as compared to non-ASD/SPAD children (4/8, 50% vs. 2/12 16.7%). In 2/4 ASD/SPAD children with seizure disorders, seizure activity was often associated with infection and is better controlled after implementation of IVIG treatment (Table 1). No subjects in the normal control and ASD/non-SPAD groups suffered from seizure disorders. As noted previously, these ASD/SPAD subjects are those with markedly worsening behavioral symptoms/cognitive skills following each immune insult [8]. These results raise the question of whether the immune abnormalities that are associated with SPAD, contribute to the clinical features observed in the ASD/SPAD children. Alternatively, it may be possible that SPAD is a part of the clinical features that develop with age in the ASD/SPAD children and are associated with immune abnormalities that affect Ab production and possibly the neuroimmune network. Interestingly, in 3/4 ASD/SPAD subjects with seizure disorders, SPAD was diagnosed several years later after the diagnosis of seizure disorders.

Routine immune workups of ASD/SPAD children did not reveal major defects of T or B cells, except for those indicating impaired antibody production typically found in SPAD patients (Table 2). Infectious complications in the ASD/SPAD children were very similar to those observed in non-ASD/SAPD children [9]. Therefore, pathogen-specific mechanisms are unlikely to explain neuropsychiatric symptoms of fluctuating behavioral symptoms/cognitive skills in the ASD/SPAD children examined in this study.

When innate immune responses were assessed, by measuring responses to a panel of TLR agonists, we observed significant differences in the ASD/SPAD children, as compared to control groups. That is, PBMCs from the ASD/SPAD children tended to produce less pro-inflammatory cytokines (TNF-α, IL-1ß, IL-6, IL-12, and IL-23). Changes were most evident in the production of IL-6. As for production of counter-regulatory cytokines, PBMCs from ASD/SPAD children revealed less spontaneous IL-10 production than normal controls. Responses to TLR agonists in the non-ASD/SPAD children differed significantly from those observed in the ASD/SPAD children as detailed in the results section and Figure 1. Only common feature found in both the ASD/SPAD and non-ASD/SPAD children was lower spontaneous production of IL-10 than normal controls (Figure 1-C).

Since ASD/SPAD children are on multiple medications for asthma, chronic rhinitis, and infection prophylaxis which included montelukast, steroid oral/nasal inhalers, anti-histamines, and azithromycin prophylaxis, these finding could be attributed to medications that they were on. However, non-ASD/SPAD children were also on multiple medications similar to those taken by ASD/SPAD children. Therefore it is unlikely that these medications are affecting the assay results. Some ASD/SPAD children were also on anti-seizure medications including levetiracetam (N = 2), valproic acid (N = 1), and lorazepam (N = 1). However, it is hard to assess if these medications can affect the assay results given low frequency of intake of these medications among the ASD/SPAD children. Two ASD/non-SPAD children with seizure disorders were also on levetiracetam and valproic acid.

IL-6 is important for B cell maturation and Ab production [16]. Our finding of impaired IL-6 production in ASD/SPAD children might be associated with development of SPAD in the ASD/SPAD children. Decreased production of IL-10 may also indicate a possibility of prolonged inflammation in ASD/SPAD children. Proinflammatory cytokines especially TNF-α, IL-1ß, and IL-6 exert important roles in mediating acute stress responses and dysregulated production of these cytokines were implicated with chronic CNS inflammation [20,21], as well as, schizophrenia [22]. Thus, lower production of these key cytokines may indicate an impairment of stress responses or the neuro-immune network in the ASD/SPAD children with fluctuating behavioral symptoms/cognitive skills.

Cytokines produced by innate immune responses greatly affect differentiation of T-helper (Th) cell subsets. IL-1ß, IL-6, and TGF-ß, when combined together, promote differentiation of Th17 cells, which in turn promote neutrophilic inflammation and anti-fungal/bacterial defense [23-25]. IL-23 sustains Th17 cells [23-25]. IL-12 promotes differentiation of Th1 cells [26]. Given decreased IL-12, IL-6, and IL-1ß production in the ASD/SPAD children, the question is raised as to whether production of T cell cytokines specific for Th cell subsets is altered in ASD/SPAD children. When we tested T cell cytokine production, our results revealed lower production of IFN-γ, Th1 cytokine, and IL-17A, Th17 cytokine, in response to PHA in the ASD/SPAD children (Figure 2-A). These results indicate a possible impairment of Th1 and perhaps Th17 responses in the ASD/SPAD children, making them more vulnerable to certain microbial infection. However, frequency of Th1 and Th17 subsets identified by intracellular cytokine expression were not altered in ASD/SPAD children. The non-ASD/SPAD children did not reveal such changes. Further studies regarding Th1/Th17 cell development will be required in the ASD/SPAD children with fluctuating behavioral symptoms and cognitive skills.

When we tested adaptive immune responses to recall Ags, we observed significantly less production of IL-10 and IL-12p40 with candida Ag, but higher increase of TGF-ß production in the ASD/SPAD children, than control groups. IFN-γ or IL-17A production with candida Ag did not differ among the study groups. Interestingly, 5 of 8 ASD/SPAD children had chronic GI inflammation often complicated by dysbiosis and/or candida enteritis with evidence of positive reactivity to candida antigen when assessed by production of IFN-γ and IL-17A production at the time of flare up. These children frequently required treatments with oral anti-fungal medications. Both IL-10 and IL-12p40 can function as regulatory factors to control inflammatory responses. Thus a decreased production of these cytokines with candida Ag may lead to persistent and excessive immune responses against candida Ag in the ASD/SPAD children.

Intracellular expression of TGF-β was lower in the ASD/SPAD children than controls (Figure 7) when stimulated with SEB or candida Ag. These findings indicate decreased frequency of TGF-β^+ ^inducible regulatory T (Treg) cells in the ASD/SPAD children, despite higher increase in TGF-ß production by ASD/SPAD PBMCs than controls. TGF-ß is produced by many lineage cells and the source of TGF-ß in the cultures of ASD/SPAD PBMCs may not be Treg cells, but other lineage cells. Such changes were not observed in non-ASD/SPAD children. In summary, studies of T cell functions indicate dysregulated T cell functions in the ASD/SPAD children, but not in the non-ASD/SPAD children. These findings may be associated with altered innate immune responses in the ASD/SPAD children.

Circulating monocytes in the PB have a short half-life and undergo spontaneous apoptosis on a daily basis [27]. In response to various differentiation factors, monocytes escape their apoptotic fate by differentiating into macrophages [27,28], which usually happens during inflammatory responses. PB monocytes are heterogeneous consisting of M1 monocytes (CD14++, CD16- cells) and M2 monocytes (CD14+, CD16+ cells) [27,29]. The majority of PB monocytes is M1 monocytes and activated M1 monocytes are recruited to the site of inflammation via chemokines (CCL2 and CCL7) [27,29]. To further extend our investigations, we conducted transcription profiling of PB monocytes in ASD/SPAD children in comparison with ASD/non-SPAD and normal controls. Over 300 genes are either up- or down-regulated in the ASD/SPAD children, in comparison with both ASD/non-SPAD and normal control groups (Table 3). Interestingly, gene expression of CCL2 and CCL7 was up-regulated in PB monocytes from the ASD/SPAD children. Recruitment of M1 monocytes has been implicated with chronic inflammatory conditions, including those in the CNS [27,30]. Thus, up-regulated expression of CCL2 and CCL7 may indicate a constant activation signal occurring in PB monocytes in the ASD/SPAD children.

The results of transcription profiling of PB monocytes also revealed enriched expression of genes in TGFBR (TGF-β receptor), NOTCH, and EGFR (epidermal growth factor receptor) signaling pathways in ASD/SPAD children, as compared to ASD/non-SPAD controls. Pathogen associated molecular patterns (PAMPs) often up-regulate NOTCH ligands and NOTCH signaling in macrophage/monocyte lineage cells [31], which can, in turn, affect production of cytokines regulating Th cell subsets [32]. NOTCH signaling is also known to affect the activation status of microglial cells and neural progenitor cells [33-35]. NOTCH and EGFR pathways are closely inter-related and involved in regulation of cell differentiation and proliferation [36,37]. TGFBR pathway activation is implicated with cell proliferation and wound healing along with its anti-inflammatory properties of TGF-ß [38]. In addition, TGF-ß pathways closely interact with NOTCH and EGFR signaling pathways [31]. Thus the findings from the transcription profiling of PB monocytes also support our assumption that ASD/SPAD monocytes are chronically activated which may be associated with the fluctuating neuropsychiatric symptoms observed in the ASD/SPAD children. Further analysis of a larger number of these children in comparison with appropriate controls will be necessary for further elucidate whether PB monocytes play a role in the medical conditions of ASD/SPAD children. In addition, follow-up of these parameters in the ASD/SPAD children longitudinally will be informative to further address their immune abnormalities in association with their above-described clinical features.

Use of IVIG for treatment of autism or its 'presumed' autoimmune co-morbid conditions, such as pediatric autoimmune neuropsychiatric disorders associated with streptococci (PANDAS), has been controversial, and clinical trials yielded conflicting results [39-41]. The controversy surrounding the effects of IVIG on ASD children is partly attributed to marked heterogeneity of ASD subjects and relatively small numbers of study subjects. In our cohorts, beneficial effects of IVIG on cognitive skills/behavioral symptoms were observed in only one of 8 ASD/SPAD subjects, indicating the complexity of ASD pathogenesis and the need for careful evaluation of the use of IVIG in ASD children, other than for antibody deficiency syndrome.

The limitation of this study is the lack of transcription profiling data in PB monocytes from the non-ASD/SPAD children, due to both limited resources and restrictions associated with the study protocol. However, to our knowledge, we have not found any literature describing similar changes in transcript profiles of PB monocytes in non-ASD/SPAD children. At least two ASD/SPAD children without fluctuation of behavioral symptoms/cognitive skills are followed in our clinic. In these children, we have not observed the immune abnormalities found in ASD/SPAD children described in this case series (unpublished observation). These results again indicate that the immune abnormalities observed in ASD/SPAD children with fluctuating behavioral symptoms/cognitive skills are not solely attributed to SPAD.

## Conclusions

In summary, our results revealed distinct clinical and immunological findings in ASD/SPAD children who reveal worsening behavioral symptoms/cognitive skills with immune insults. These immune abnormalities are not shared by either ASD/non-SPAD or non-ASD/SPAD children. Thus these abnormalities are likely to be more specific for this subset of ASD children and may be associated with their worsening behavioral symptoms/cognitive skills with immune insults. Development of SPAD in these ASD children may also be a part of their clinical spectrum associated with these immune abnormalities.

## Abbreviations

Abbreviations used are as follows: AA: African American; Ab: antibody; Ag: antigen; ASD: autism spectrum disorders COM; chronic otitis media CRS: chronic rhinosinusitis; CVID: common variable immunodeficiency; FA: food allergy; FPIES: food protein induced enterocolitis syndrome; IVIG: intravenous immunoglobulin; IL: interleukin; PB: peripheral blood; PBMCs: peripheral blood mononuclear cells; PDD-NOS: pervasive developmental disorder, not otherwise specified; PE tube: pressure equalizing tube; SEB: staphylococcal enterotoxin B; SPAD: specific polysaccharide antibody deficiency; sTNFRII: soluble TNF receptor II; Th: T-helper; TGF: transforming growth factor; TNF: tumor necrosis factor.

## Competing interests

The authors declare that they have no competing interests.

## Authors' contributions

HJ was responsible for the study design, recruitment of the study subject, collection of clinical information and blood samples, and analysis of the data of cytokine production assays and flow cytometry. She was also mostly responsible for preparation of this manuscript. LG conducted most of cytokine production assays as well as staining cells for flow cytometry and assisted the first author for data analysis. She was also responsible for PB monocytes purification and preparation of total RNA from PB monocytes. DLS was mostly responsible for conducting transcription profiling of PB monocytes.

GAT was responsible for experimental design of transcription profiling and analysis of the data of transcription profiling as well as manuscript preparation associated with transcription profiling. All the authors read and approved the final manuscript.

## Authors' information

Harumi Jyonouchi, M.D.: She is a board certified in Pediatrics and Allergy/Immunology and currently serves as an Associate Professor of Pediatrics, UMDNJ-NJMS. She has conducted several clinical studies addressing relationship of delayed type food allergy (FA) with GI symptoms in ASD children as well as immune abnormalities in a subset of ASD children with distinct clinical characteristics.

Gokce A. Truner, M.D., Ph.D.: He is a molecular geneticist and currently is an Assistant Professor at the Institute of Genomic Medicine, UMDNJ-NJMS. He has been involved in clinical studies of CGH, transcription profiling, and microRNA profiling in patients with various medical conditions including children with mental retardation and ASD.
